# MiR-223 regulates autophagy associated with cisplatin resistance by targeting FBXW7 in human non-small cell lung cancer

**DOI:** 10.1186/s12935-020-01284-x

**Published:** 2020-06-19

**Authors:** Hui Wang, Jiabin Chen, Shufen Zhang, Xiaoxiao Zheng, Shangzhi Xie, Jiayan Mao, Ying Cai, Xuemei Lu, Liqiang Hu, Jian Shen, Kequn Chai, Wei Chen

**Affiliations:** 1 Zhejiang Chinese Medical University, 548 Binwen Road, Binjiang District, Hangzhou, 310053 Zhejiang China; 2 Cancer Institute of Integrated Traditional Chinese and Western Medicine, Zhejiang Academy of Traditional Chinese Medicine, Tongde Hospital of Zhejiang Province, No.234, Gucui Road, Hangzhou, 310012 Zhejiang China

**Keywords:** MiR-223, Chemoresistance, Non-small cell lung cancer (NSCLC), Autophagy, FBXW7

## Abstract

**Background:**

Cisplatin is widely used as a first-line treatment for non-small cell lung cancer (NSCLC), but chemoresistance remains a major clinical obstacle for efficient use. As a microRNA, miR-223 was reported to promote the doxorubicin resistance of NSCLC. However, whether miR-223 is also involved in cisplatin resistance of NSCLC and the mechanism miR-223 involved in drug resistance is unclear. Accumulated evidence has shown that abnormal autophagy is associated with tumor chemoresistance. The study aimed to study the role of miR-223 on cisplatin sensitivity in NSCLC and uncover the potential mechanisms.

**Methods:**

NSCLC cells transfected with mimic or inhibitor for miR-223 was assayed for chemoresistance in vitro. MiR-223 expression was assessed by quantitative real-time PCR (qRT-PCR). Western blot were used to study the expression level of F-box/WD repeat-containing protein 7 (FBXW7) and autophagy-related protein. The effect of miR-223 on cisplatin sensitivity was examined by using CCK-8, EdU assays and Autophagic flux assay. Luciferase assays, EdU assays and small interfering RNA were performed to identify the targets of miR-223 and the mechanism by which it promotes treatment resistance. Xenograft models were established to investigate the effect of mir-223 on cisplatin sensitivity.

**Results:**

In the present study, we found that the level of miR-223 was significantly positively correlated with cisplatin resistance. MiR-223 overexpression made NSCLC cells resistant to cisplatin treatment. We further found that autophagy mediated miR-223-mediated cisplatin resistance in NSCLC cells. Further mechanistic research demonstrated that miR-223 directly targeted FBXW7. The overexpression of miR-223 could inhibit the level of FBXW7 protein expression, thus promoting autophagy and making NSCLC cells resistant to cisplatin. Finally, we confirmed the increased effect of cisplatin sensitivity by miR-223 Antagomir in xenograft models of NSCLC.

**Conclusions:**

Our results demonstrate that miR-223 could enhance autophagy by targeting FBXW7 in NSCLC cells. Inhibition of autophagy by miR-223 knockdown provides a novel treatment strategy to alleviate cisplatin resistance in NSCLC.

## Background

Although substantial effort has been paid to improving patient survival, lung cancer remains a leading cause of cancer-related mortality worldwide [1, 2]. Non-small cell lung cancer (NSCLC) accounted for approximately 80––85% of all lung cancer cases, with an overall 5-year survival rate of < 20% [3]. In addition, cisplatin-based traditional chemotherapy and molecular targeting drug therapy have greatly improved the prognosis and quality of life in NSCLC patients [4, 5]. However, drug resistance is a vital bottleneck, which limits the effect of chemotherapy in most NSCLC patients [6, 7]. Therefore, elucidating the molecular mechanisms underlying drug resistance in NSCLC is critical.

MicroRNA (miRNA) is an endogenous non-coding RNA between 18 and 25 nucleotides, has been shown to regulate mRNA and protein expression by binding to the 3′-untranslated region (3′-UTR) of their target genes. Several previous studies on miRNA in NSCLC have been exerted and a series of miRNAs have been found to be involved in the development of NSCLC drug resistance [8, 9]. Moreover, abnormal miR-223 expression has been detected in various cancers [10–15]. In NSCLC, previous studies described a controversial role of miR-223, functioning as either a tumor suppressor or an oncogene. In addition, Huang et al. reported that miR-223 may promote malignant phenotypes of lung cancer in A549 cells via activation of the NF-κB signaling pathway [16]. Liang et al. reported that platelet-secreted microvesicles (P-MVs) can promote lung cancer cell invasion via targeting the tumor suppressor, EPB41L3 [17]. In contrast, Zhou et al. reported that miR-223 inhibits tumor development of NSCLC and sensitizes A549 cells to gefitinib via targeting E2F1 [18]. Moreover, miR-223 was found to enhance the sensitivity of NSCLC cells to erlotinib by targeting the insulin-like growth factor-1 receptor [19]. However, another study showed that miR-223 could induce doxorubicin resistance through targeting F-box/WD repeat-containing protein 7 (FBXW7)-mediated epithelial mesenchymal transition in NSCLC cells [20]. Thus, additional studies are essential to further uncover the role of miR-223 in the development and drug resistance in NSCLC.

Autophagy is vital biological process required for the maintenance of cellular biosynthesis, growth, and differentiation. Accumulating evidence has demonstrated that the abnormal activation of autophagy is an important factor involved in the process of chemoresistance [21, 22]. Moreover, recent studies indicate that miRNAs are frequently dysregulated in chemoresistant lung cancers, in which they have been shown to target autophagy-related genes or modulators. For example, miR-200b was shown to regulate autophagy associated with chemoresistance in human lung adenocarcinoma by targeting ATG12 [23]. In addition, the downregulation of miR-24-3p could induce etoposide (VP16)-cisplatin resistance in small-cell lung cancer by targeting ATG4A [24]. However, the relationship between miR-223 and autophagy in cancer remains poorly understood.

In the present study, we aimed to study the role of miR-223 on cisplatin resistance in NSCLC and uncover the potential mechanisms. In the present study, we found that miR-223 could enhance cisplatin resistance in NSCLC by targeting FBXW7 and upregulating autophagy. Thus, we aimed to identify the pro-chemoresistant role of miR-223 in NSCLC cells in vitro.

## Materials and methods

### Cell lines and reagents

Human NSCLC A549, NCI-H358, and NCI-H1299 cells were purchased from the ATCC (Manassas, VA, USA) and cultured in RPMI 1640 medium containing 10% fetal bovine serum. PC9 was purchased from the Chinese Academy of Science Cell Bank (Shanghai, China) and cultured in DMEM medium containing 10% fetal bovine serum. All cell lines were cultured at 37 °C in a humidified atmosphere (95% air and 5% CO_2_). Cisplatin, rapamycin, and chloroquine were purchased from Sellerck (Huston, TX, USA).

### siRNA and transfection

The miRNA mimics, miRNA inhibitors, and FBXW7 siRNA were synthesized by GenePharma (Shanghai, China). The sequences of primers were placed in Additional file 1: Table S1. Cells were transfected using Lipofectamine 3000 (Invitrogen, USA), according to the manufacturer’s protocol.

### Cell viability assay

Cells were seeded into 96-well plates (4 × 10^3^ cells/well) directly or 24 h after transfection. After treatment with different concentrations of cisplatin combinations for 48 h, cell viability was examined using a commercial CCK-8 kit (Dojindo, Kumamoto, Japan) according to the manufacturer’s protocol, and the absorbance was determined at 450 nm using an MRX II microplate reader (Dynex, Chantilly, VA, USA).

### Western blot

Cells were washed with PBS, harvested in ice-cold PBS, centrifuged at 2000 rpm at 4 °C, and lysed in RIPA buffer; protein concentrations were determined with a BCA kit (Pierce, Rockford, IL, USA). An equal amount of cell lysate for each condition was subjected to SDS-PAGE, transferred onto nitrocellulose members, and analyzed. The primary antibodies against FBXW7, LC3-I, LC3-II, SQSTM1, and β-actin were purchased from Abcam (Cambridge, USA) and used at a concentration of 1:1000. The corresponding secondary antibody was also obtained from Abcam and used at a concentration of 1:5000.

### Real-time quantitative PCR (qRT-PCR)

Total RNA was extracted using Trizol (Takara, Japan) reagent. Reverse transcription and qRT-PCR were conducted using a SYBR Prime Script™ miRNA RT-PCR kit (Takara, Japan,) according to the manufacturer’s instructions. The level of miR-223 expression was normalized to *U6* RNA. SYBR Premix Ex Taq (Takara, Japan) was also used to detect the level of FBXW7 mRNA. The sequences of primers were placed in Additional file 1: Table S1. Relative mRNA expression was normalized to β-actin. Data were analyzed using the 2ΔΔCt method.

### EdU assay

Proliferation of the NSCLC cell lines was determined using a Click-iTEdU Imaging Kit (Invitrogen; Carlsbad, CA, USA) according to the manufacturer’s protocol. Briefly, cells were treated with different conditions for 24 h, and 10 μM EdU was added for 2 h before fixation and permeabilization. Cell nuclei were stained with Hoechst 33342 (Invitrogen) at a concentration of 5 μg/mL for 30 min.

### Luciferase assays

The 293T cells were co-transfected with wild-type or mutant FBXW7 3′-UTR plasmid (Promega) as well as miR-223-3p mimics or miR-223-3p inhibitor (Ribo) using Lipofectamine 2000 (Invitrogen). Cell lysates were harvested 48 h after transfection and then firefly and Renilla luciferase activities were measured by a dual luciferase reporter assay kit according to the manufacturer’s protocol. Renilla luciferase activity was used for normalization.

### Autophagic flux assay

A549 and NCI-H1299 cells stably transfected with RFP-GFP-LC3 adenovirus were subjected to different treatments. After 48 h, the cells were fixed with 4% paraformaldehyde (Sigma, USA) and photographed using a laser confocal fluorescence microscope. Cells were detected by the expression of green (GFP) or red (RFP) fluorescence. Autophagosomes were characterized by yellow puncta and autolysosomes based on only red puncta in the merged images. Autophagic flux was determined by an increased percentage of only red puncta in the merged images. A total of 300 cells were randomly selected to be counted and the number of autophagosomes and autolysosomes were averaged.

### Flow cytometry assay

Cells were treated with cisplatin (IC50) for 48 h. The cells were stained with the Annexin-V and 7AAD according to the manufacturer’s protocol. The rate of apoptosis was determined by flow cytometry.

### Immunohistochemistry and terminal uridine deoxynucleotidyl transferase dUTP nick-end labeling (TUNEL) assay

The immunohistochemistry assay of Ki-67 was performed on 4 μm of a thickness of paraffin-embedded mouse tissue sections. The sections were then incubated with the anti-Ki-67 antibody (1:500, Abcam) at 4 °C overnight. Then the primary antibodies were detected with an HRP-linked secondary antibody (Abcam) and developed using a 3ʹ-diaminobenzidine substrate kit. The positively stained cells were defined as those with brown nuclei, and the percentage of positive tumor cells was determined. For apoptosis detection, apoptosis of the tumor tissues was determined with TUNEL assay using the In Situ Cell Death Detection Kit (Roche) according to the manufacturer’s protocol. The apoptotic cells were observed under a light microscope.

### Tumor xenograft studies

Animal experiments were conducted in compliance with the Guide for the Care and Use of Animal Ethics Committee of Zhejiang Chinese Medicine University (Hangzhou, China). 5 × 10^6^ prepared A549 cells were injected subcutaneously into the right axillary fossa. Tumor length (L) and width (W) were measured and tumor volumes were calculated using the formula (L × W^2^)/2. When tumor volumes reached 100 mm^3^, the mice were randomly divided into four groups (n = 3), as followed: negative control, 5 mg/kg cisplatin, 8 nmol antagomiR-223, or cisplatin combined with antagomiR-223. Cisplatin and antagomiR-223 were administered by intraperitoneal and intratumor injection, respectively, every 2 days for 2 weeks. All the mice were euthanized.

### Statistical analysis

Data are presented as the mean ± SD from three independent experiments and analyzed by a two-tailed Student’s *t*-test using SPSS 18.0. Differences were considered significant at a value of *P *< 0.05.

## Results

### The level of miR-223 was positively correlated with cisplatin resistance in NSCLC cells

**Fig. 1:**
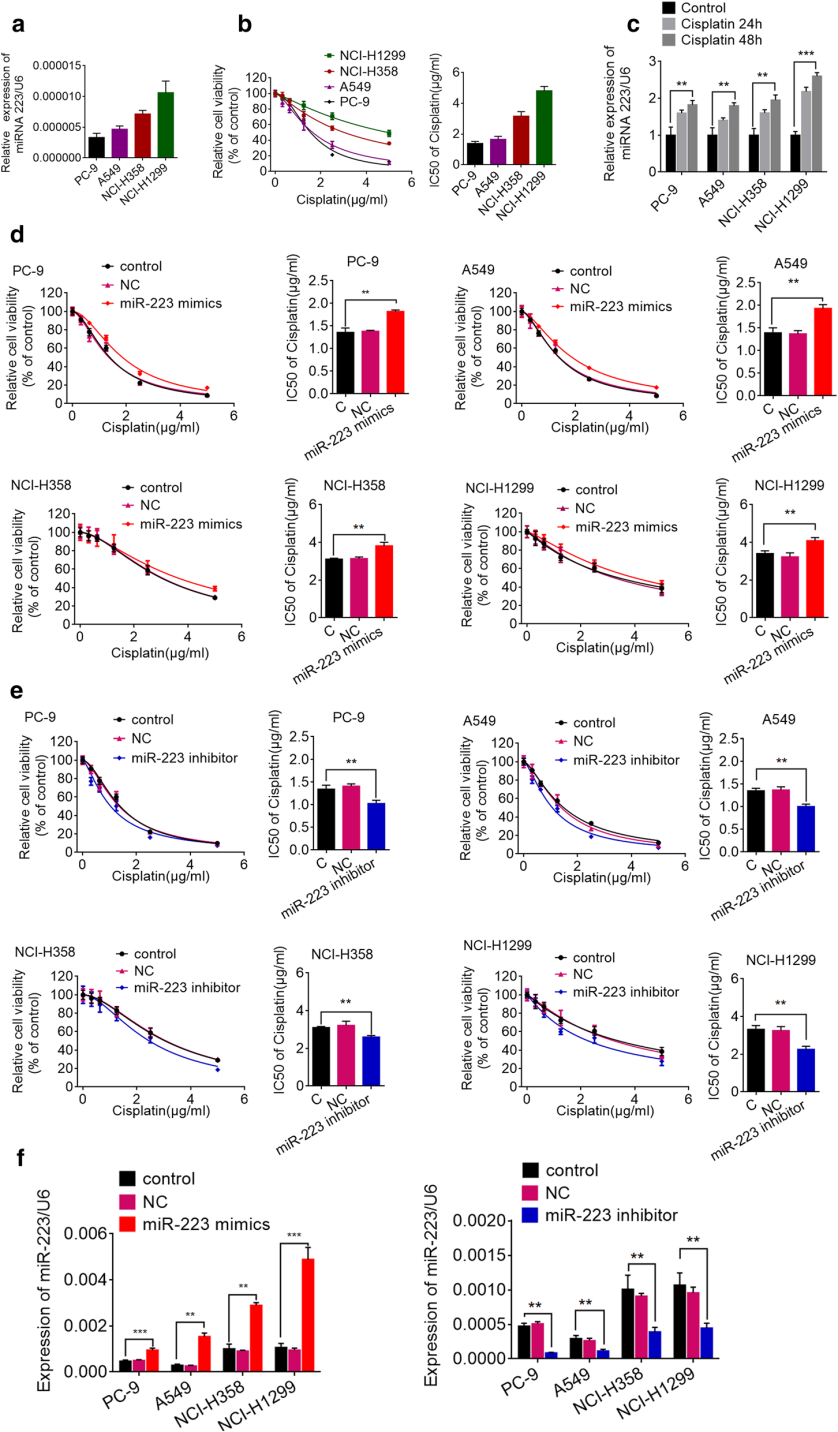
Forced miR-223 expression enhanced the cisplatin resistance of NSCLC cells. **a** Expression of miR-223 in NSCLC cell lines were detected by qRT-PCR. U6 was used as the internal reference. **b** Cell viability of NSCLC cell lines treated with different concentrations of cisplatin with a CCK-8 assay and IC50 value was calculated. **c** Expression of miR-223 in NSCLC cell lines were detected by qRT-PCR after cisplatin treatment. **P < 0.01; ***P < 0.001 compared with the control. **d**, **e** NSCLC cells were transfected with an miR-223 mimic, miR-223 inhibitor, or the scrambled control. After 48 h post-transfection, different concentrations of cisplatin were added and cell viability was determined with a CCK-8 assay after 24 h. **P < 0.01 vs. the control. **f** Real-time PCR was used to validate the efficiency of the miR-223 inhibitor or miR-223 mimic. **P < 0.01; ***P < 0.001 compared with the control

To investigate the biological role of miR-223 in NSCLC cisplatin resistance, we first quantified the level of miR-223 in four NSCLC cells. Our results revealed that the level of miR-223 was the highest in NCI-H1299 cells and lowest in PC-9 cells (PC-9 < A549 < NCI-H358 < NCI-H1299; Fig. 1a). Next we performed a CCK-8 assay to examine the viability of NSCLC cell lines exposed to cisplatin for 48 h. Interestingly, the cell viability in each cell line represented an positive trend with the level of miR-223 (Fig. 1b); NSCLC cells with high miR-223 levels were more resistant to cisplatin. Moreover, miR-223 expression in cisplatin-treated NSCLC cells was significantly enhanced compared with control (Fig. 1c). Therefore, miR-223 expression may be involved in cisplatin resistance in NCSLC cells and could be induced in vitro during cisplatin therapy.

### MiR-223 regulates NSCLC cell sensitivity to cisplatin

To further investigate the role of miR-223 on cisplatin resistance, NSCLC cells were transfected with a miR-223 mimics, inhibitor or corresponding negative control (NC), respectively. And the effect on cellular viability was assessed followed by cisplatin treatment after transfection. As shown in Fig. 1d, transfection with the miR-223 mimics gave rise to a marked decrease in cisplatin sensitivity in the NSCLC cells. In contrast, as shown in Fig. 1e, treatment with the miR-223 inhibitor resulted in a marked increase in cisplatin sensitivity. Transient miR-223 transfection efficiency is presented in Fig. 1f. These results indicate that the forced expression of miR-223 promoted cisplatin resistance in NSCLC cells.

### Autophagy is involved in miR-223 mediated cisplatin resistance in NSCLC cells

**Fig. 2:**
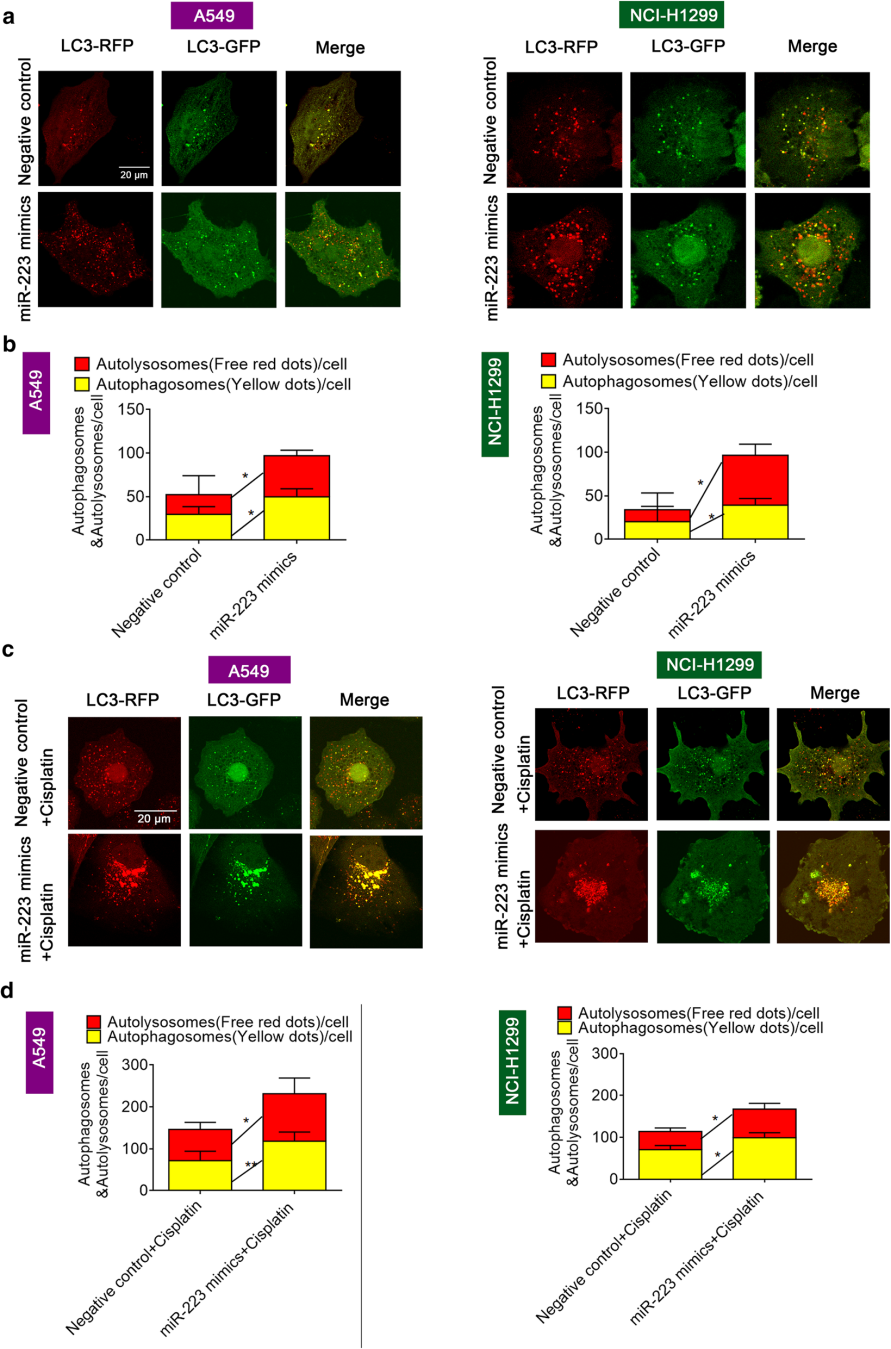
MiR-223 overexpression promoted autophagy in NSCLC cells. **a**, **b** Autophagy flux was examined in A549 and NCI-H1299 cells transfected with the miR-223 mimics or miR-control using a tandem adenovirus RFP-GFP-LC3 construct. The number of autophagosomes (yellow dots) and autolysosomes (red-only dots) were assessed. *P < 0.05 compared with Negative control. **c**, **d** Autophagy flux was examined in A549 and NCI-H1299 cells transfected with the miR-223 mimics or miR-control following treatment with the IC_50_ of cisplatin. The number of autophagosomes (yellow dots) and autolysosomes (red-only dots) were assessed. *P < 0.05; **P < 0.01 compared with Negative control + cisplatin

Since autophagy has been found to play a vital role in the development of chemoresistance in tumor cells [25], we hypothesized that miR-223 may help increase autophagy in NSCLC cells. To verify the hypothesis, we overexpressed miR-223 by transfecting NSCLC cells with the miR-223 mimics and then tested the autophagic flux using a tandem RFP-LC3-GFP construct. Our results demonstrated that both of the number of green-red (yellow) dots (representing autophagosomes) and the number of red-only dots (representing autolysosomes) were significantly increased following miR-223 overexpression, and the percentage of autolysosomes was increased, indicating enhanced autophagy (Fig. 2a, b).

We next explored whether autophagy is involved in miR-223-mediated cisplatin resistance. Firstly, we detected the role of cisplatin in regulating autophagy. A Western blot analysis showed that cisplatin treatment increased the level of LC3-I to LC3-II conversion and decreased the level of SQSTM1 expression, indicating enhanced autophagy (Additional file 2: Fig. S1). Secondly, we determined the level of autophagy in NSCLC cells treated with the miR-223 mimics compared to the negative control transfection following cisplatin co-treatment. We found that the cisplatin induced autophagy flux was enhanced by the miR-223 mimic, and the percentage of red-only dots indicative of autolysosomes significantly increased (Fig. 2c, d). In addition, we detected the cell apoptosis by flow cytometry assay. The results showed that miR-223 overexpression inhibited the cisplatin-induced apoptosis (Additional file 3: Fig. S2).

**Fig. 3:**
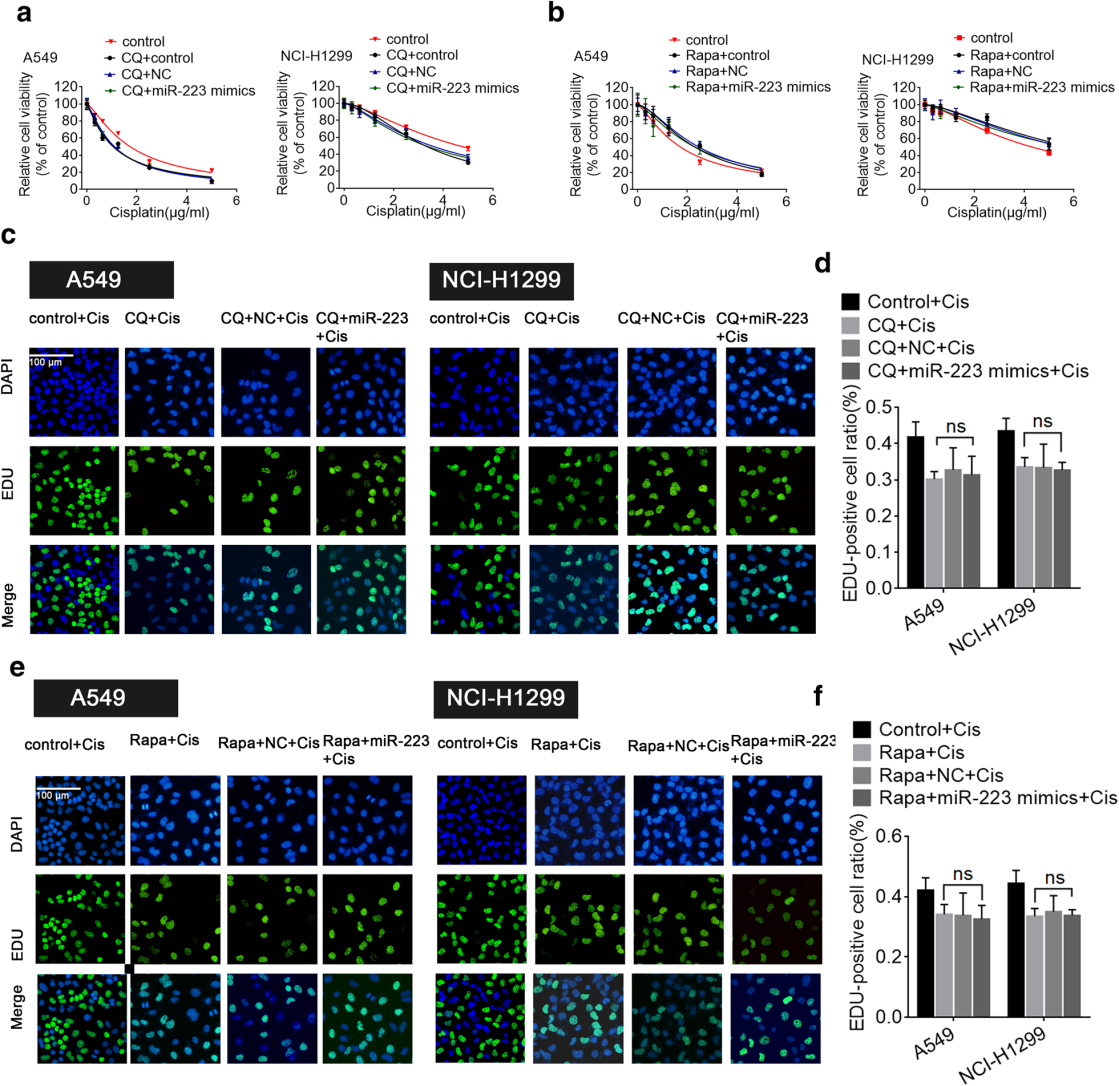
Blocking autophagy reversed miR-223-mediated cisplatin resistance. **a** Cell viability of A549 and NCI-H1299 cells cultured in different concentrations of cisplatin following 10 μM chloroquine and transfection with either the miR-223 mimics or miR-control. **b** Cell viability of A549 and NCI-H1299 cells cultured in different concentrations of cisplatin following treatment with 100 nM rapamycin and transfection with either the miR-223 mimics or miR-control. **c**, **d** Cellular proliferation of A549 and NCI-H1299 cells cultured in the IC50 of cisplatin following treatment with 10 μM chloroquine or the control component and transfection with the miR-223 mimics or miR-control transfection detected using an EdU assay. **e**, **f** Cellular proliferation of A549 and NCI-H1299 cells cultured in the IC50 of cisplatin following 100 nM rapamycin or the control component and transfected with either the miR-223 mimics or miR-control detected using an EdU assay. Photomicrographs and bar graphs depicted the EdU staining and relative EdU-positive ratio, respectively

Finally, a well-established autophagy inducer, rapamycin (RAPA), and autophagy blocker, chloroquine, were used to induce or inhibit autophagy, respectively. Chloroquine could enhance the sensitivity of NSCLC cells to cisplatin and increase SQSTM1 levels, a selective marker of autophagy while RAPA had the opposite effect (Additional file 4: Fig. S3). When NSCLC cells pretreated with CQ or RAPA were transfected with either the miR-223 mimics or negative miR-control, the cell viability results revealed that no matter autophagy was blocked or excessively induced, no significant difference was observed in CQ/RAPA group with or without miR-223 mimics (Fig. 3a, b). Consistent with the CCK-8 results, the Edu assay confirmed that the autophagy process was involved in miR-223-mediated cisplatin resistance in NSCLC cells (Fig. 3c–f). Taken together, these data indicate that miR-223 may increase cisplatin resistance by up-regulating autophagy.

### FBXW7 is a direct target of miR-223

**Fig. 4:**
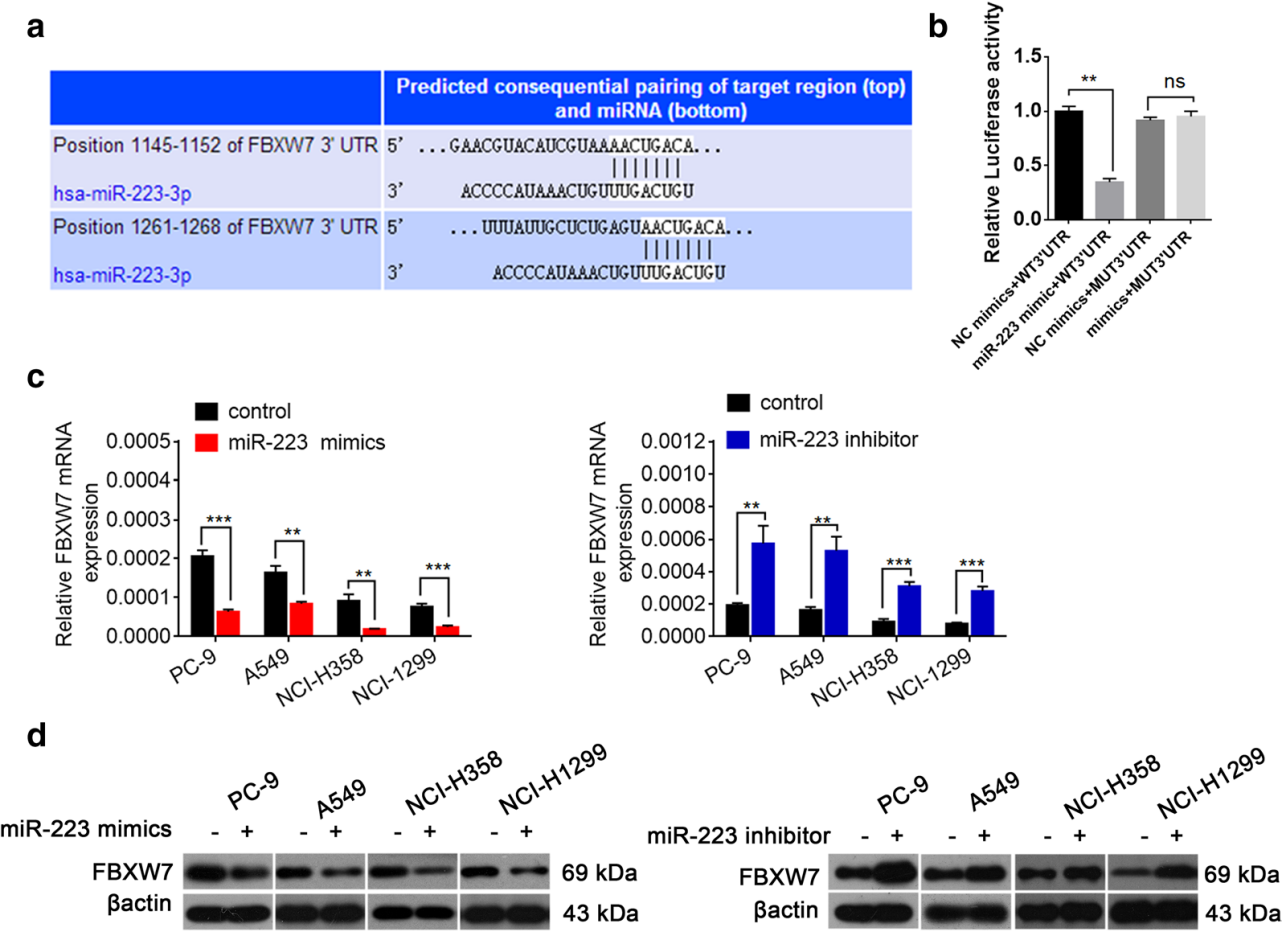
miR-223 directly targets FBXW7 in NSCLC cells. **a** The predicted binding sequences of miR-223 in the 3′UTR of FBXW7 by TargetScan. **b** miR-223-3p mimics suppressed the luciferase activity of the wild-type but not mutant of FBXW7 reporter in 293T cells. **P < 0.01 compared with NC mimic + WT 3′UTR group. **c** Quantitative RT-PCR analyses of the effect of transient transfection with either the miR-223 mimic, miR-223 inhibitor, or negative control on the level of FBXW7 mRNA expression in NSCLC cells. *P < 0.05; **P < 0.01; ***P < 0.001 compared with control. **d** Quantification of FBXW7 protein expression in NSCLC cells transfected with either the miR-223 mimic, miR-223 inhibitor, or negative control by Western blot

To reveal the mechanism by which autophagy is enhanced by miR-223, we searched for potential target genes using targetscan, a bioinformatic database, and found that miR-223 was predicted to bind with 3′UTR regions of FBXW7 mRNA (Fig. 4a). Next we performed the luciferase activity to verify it, The results showed that the relative luciferase activity of the FBXW7-Wt plasmid was significantly suppressed after co-transfection with the miR-223-3p mimic. In contrast, this effect was not detected in the plasmid carrying the FBXW7-Mut (Fig. 4b). A qRT-PCR analysis found that the forced expression of miR-223 suppressed the level of FBXW7 mRNA in NSCLC cells. Conversely, the inhibition of miR-223 increased the level of FBXW7 mRNA (Fig. 4c). Moreover, Western blots showed that the overexpression of miR-223 inhibited the level of FBXW7 protein expression and miR-223 suppression up-regulated the level of FBXW7 protein (Fig. 4d). These results demonstrate that miR-223 directly targeted FBXW7 in NSCLC cells.

### MiR-223 increases autophagy and promotes cisplatin resistance by targeting FBXW7 in NSCLC cells

**Fig. 5:**
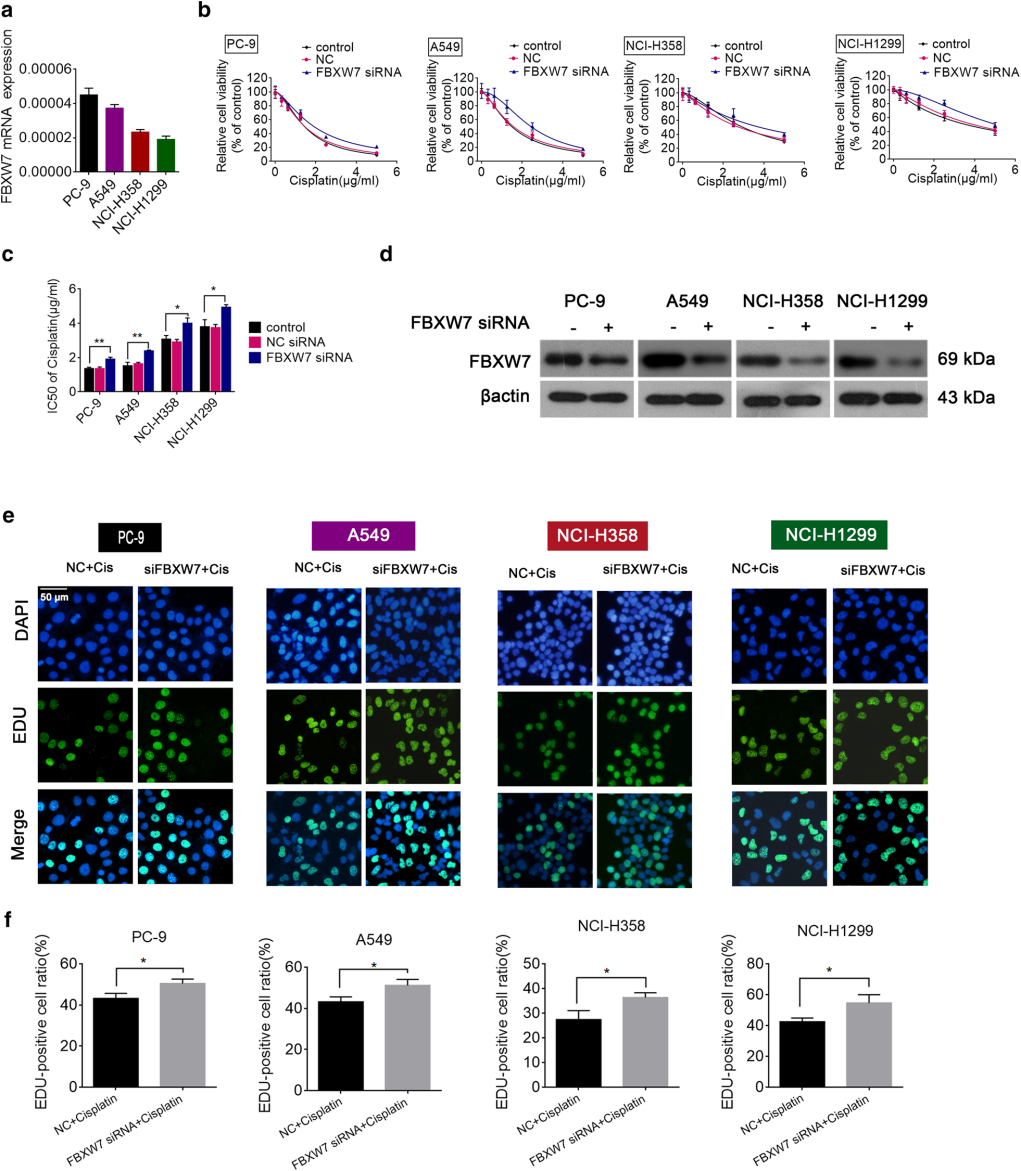
FBXW7 mediates miR-223 induced cisplatin-resistance and high levels of autophagy. **a** Quantitative RT-PCR analyses of the level of FBXW7 mRNA in NSCLC cells. **b**, **c** NSCLC cells were transfected with FBXW7 siRNA or the scrambled control. At 48 h post-transfection, different concentrations of cisplatin were added and cell viability was determined with a CCK-8 assay 24 h later and IC50 value was calculated. *P < 0.05; **P < 0.01 compared with control. **d** Immunoblot analyses of the effect of FBXW7 siRNA on NSCLC cells. **e**, **f** The cellular proliferation of NSCLC cells cultured in the IC50 of cisplatin following transfection with FBXW7 siRNA or the scrambled control detected via an Edu assay. Photomicrographs and bar charts depicted EdU staining and the relative EdU-positive ratio, respectively. *P < 0.05 compared with the NC + cisplatin group

Based on previous studies that suggested that FBXW7 regulates autophagy in several diseases and is a chemoresistance-related gene [26–28], we hypothesized that FBXW7 may mediate miR-223-induced enhanced autophagy and cisplatin resistance in NSCLC cells. To confirm this hypothesis, we first examined the level of FBXW7 mRNA and protein expression in NSCLC cells. Our results revealed that the level of FBXW7 expression level was inversely related to the level of miR-223 in NSCLC cells (Fig. 5a, Additional file 5: Fig. S4). Moreover, the knockdown of FBXW7 increased the resistance of NSCLC cells to cisplatin, which was consistent with the miR-223 overexpression findings (Fig. 5b, c). Next, the interfering efficiency of FBXW7 siRNA was determined (Fig. 5d). The cellular proliferation findings obtained from the Edu assay confirmed this result (Fig. 5e, f).

**Fig. 6:**
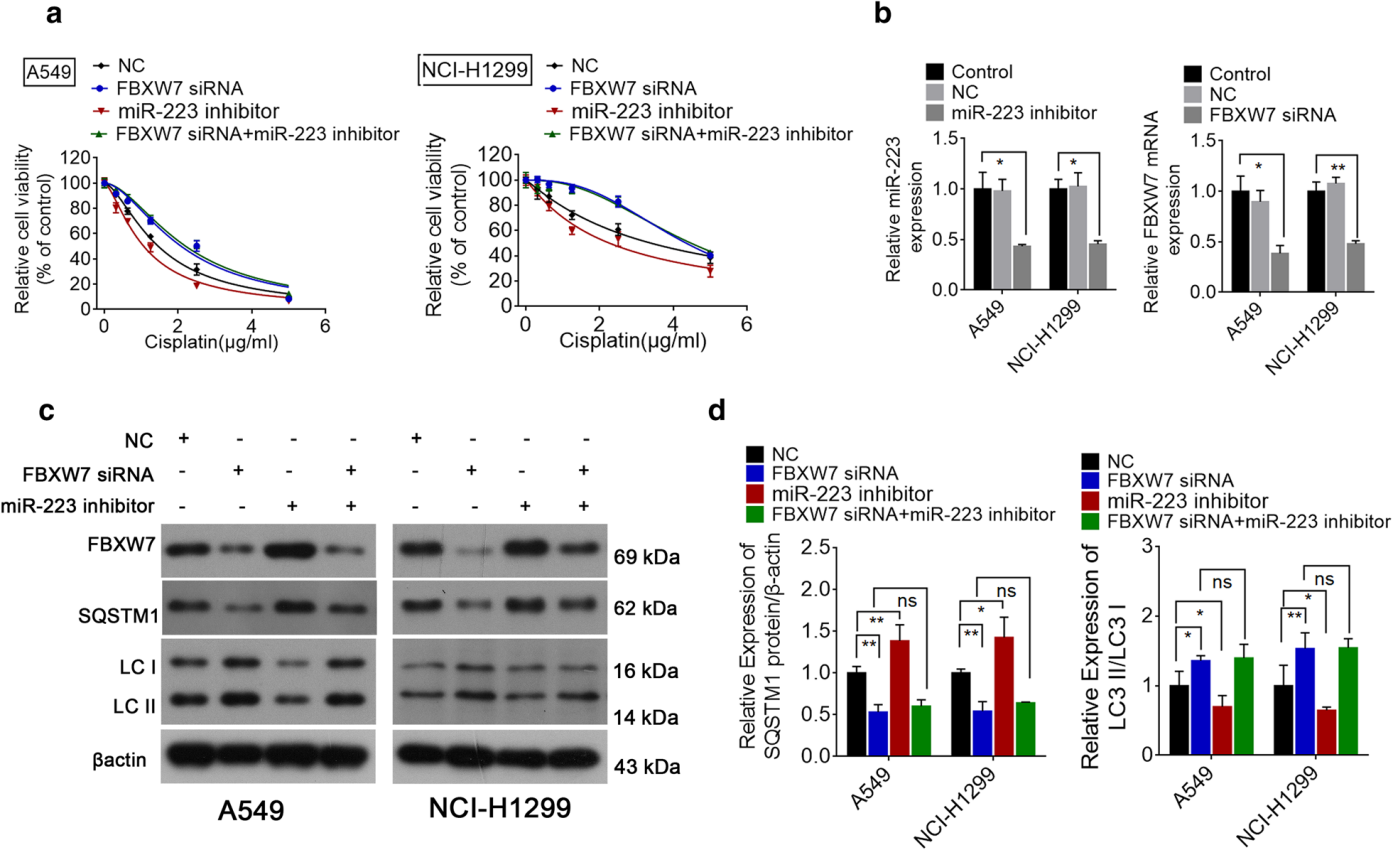
miR-223 induced cisplatin resistance by suppressing FBXW7. **a** Cell viability of A549 and NCI-H1299 cells cultured in different concentrations of cisplatin following treatment with the miR-223 inhibitor plus transfection with FBXW7 siRNA or FBXW7 siRNA alone. *P < 0.05; **P < 0.01 vs. control. **b** Real-time PCR was used to validate the efficiency of the miR-223 inhibitor or FBXW7 siRNA. **c**, **d** Western blot of FBXW7, LC3-I/II, and SQSTM1 in NSCLC cells treated with the miR-223 inhibitor plus transfection with FBXW7 siRNA, FBXW7 siRNA alone, or the scrambled control. *P < 0.05; **P < 0.01 vs. NC

To further confirm that miR-223 exerts its influence primarily by directly targeting FBXW7, we employed rescue experiments by co-transfecting NSCLC cells with FBXW7 siRNA and the miR-223 inhibitor. We found that the miR-223-inhibition induced cytotoxic effects of cisplatin were eliminated by a knockdown of FBXW7 (Fig. 6a). The transfection efficiency is presented in Fig. 6b. The results from the Western blot analysis showed that the miR-223 knockdown led to the inhibition of autophagy, which was reversed by specific the FBXW7 siRNA (Fig. 6c, d). These results demonstrate that miR-223 directly targets FBXW7 to mediate cisplatin resistance and induce autophagy.

### MiR-223 inhibition enhances the efficacy of cisplatin for NSCLC in vivo

**Fig. 7:**
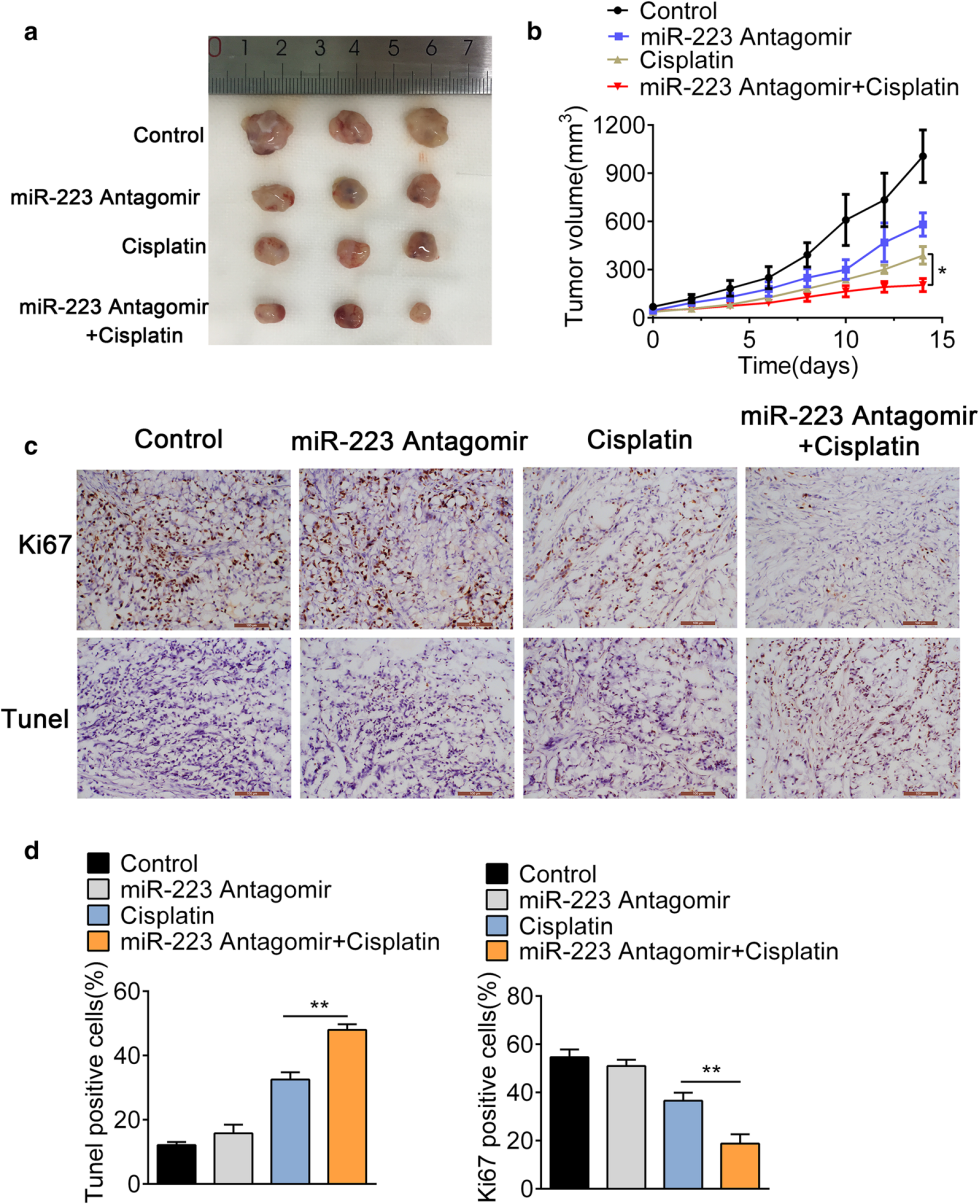
MiR-223 inhibition enhances the efficacy of cisplatin for NSCLC in vivo. **a** Representative tumour images (n = 3 per group) were shown. The tumors were treated with control, cisplatin, miR-223 Antagomir, or cisplatin plus miR-223 Antagomir. **b** Growth curves of xenograft tumors treated with control, cisplatin, miR-223 Antagomir, or cisplatin plus miR-223 Antagomir. *P < 0.05 vs cisplatin. **c**, **d** Representative Ki-67 staining and TUNEL of apoptosis images in the treatment groups. **P < 0.01 vs. cisplatin

To investigate the effects of miR-223 on cisplatin sensitivity, xenograft models were established via subcutaneous injection of A549 cells. The mice were treated with normal saline alone, cisplatin alone, miR-223 Antagomir alone, or cisplatin plus miR-223 Antagomir. Tumor growth was found no significant difference between the miR-223 Antagomir alone and the control group, while combined treatment led to significant inhibition of tumor growth compared with cisplatin alone (Fig. 7a, b). Ki-67 staining exhibited significantly decreased tumor cell proliferation rates in the cisplatin plus miR-223 Antagomir group compared to the cisplatin alone group (Fig. 7c, d); TUNEL assay showed highest tumor cell apoptosis in the cisplatin plus miR-223 Antagomir group (Fig. 7c, d). Taken together, these data confirmed that miR-223 inhibition enhances the efficacy of cisplatin for NSCLC in vivo.

## Discussion

The cytotoxic-based platinum compound, cisplatin (cisplatin), has been commonly used as a first line treatment in NSCLC for decades. However, drug resistance to cisplatin in cancer cells remains a substantial challenge to a favorable prognosis [6]. The development of cisplatin resistance is a complex multifactorial process, involving reactive oxygen species (ROS), the aberrant expression of microRNAs, ATP-binding cassette (ABC) transporter effusion, and abnormal signaling pathways [29, 30]. Accumulating evidence has demonstrated that autophagy is an essential pathway for cellular homeostasis and many studies have demonstrated that autophagy is involved in chemoresistance [31, 32]. Additionally, autophagy inhibitors have been explored as a means to sensitize chemoresistant cells to chemotherapy in clinical trials [33]. Recently, the regulation of autophagy by miRNAs has been shown to be a potentially effective strategy to reduce cancer cell chemoresistance [34]. Regarding NSCLC, while some studies have revealed a potential role of miRNAs and autophagy in cisplatin resistance in NSCLC [35, 36], no investigations have verified the ability of miR-223 and FBXW7 to directly impact the regulation of autophagy and cisplatin resistance in NSCLC cells. Therefore, additional studies are required to elucidate the relationship between miRNAs, autophagy, and cisplatin resistance in NSCLC. In the present study, we describe a novel mechanism by which miR-223 mediates cisplatin resistance by inhibiting FBXW7 to promote cisplatin-induced autophagy in NSCLC.

Increasing evidence has shown that miR-223 can function as either a cancer inducer or a tumor suppressor and is correlated with chemoresistance or chemosensitivity depending on the tumor type. In human gastric cancer, miR-223 was found to promote cisplatin resistance in human gastric cancer cells by regulating cell cycle through targeting FBXW7 [37]. In hepatocellular carcinoma, miR-223 was reported to modulate multidrug resistance via the downregulation of ABCB1 [38]. Moreover, in Glioblastoma, miR-223 was found to promote temozolomide chemoresistance in glioblastoma multiforme cells by targeting paired box 6 signaling [39]. However, the role of miR-223 on chemoresistance is inconsistent regarding NSCLC. Zhang et al. reported that increasing miR-223 expression could induce cell resistance to erlotinib in HCC827 cells [40]. In contrast, Han et al. reported that miR-223 could reverse the resistance of EGFR-TKIs through the IGF1R/PI3K/Akt signaling pathway in NSCLC [41]. In addition, Zhou et al. reported that miR-223 could sensitize cancer cells to gefitinib by targeting E2F1 [18]. Therefore, additional studies are required to further elucidate the role of miR-223 in NSCLC chemoresistance. In the present study, we showed that miR-223 can regulate cisplatin sensitivity in NSCLC by autophagy, indicating that miR223 may be mediate cisplatin resistance by autophagy. However, of these mechanisms of cisplatin resistance, the altered expression of MDR-related genes is the most common [42, 43]. Maybe mir223-mediated cisplatin resistance is correlated with altered expression of MDR-related genes.

FBXW7, also known as FBW7, is a F-box-containing protein in the SCF E3 ligase complex, which functions in phosphorylation-dependent ubiquitination [44]. Moreover, FBXW7 has been identified as a tumor suppressor gene in several cancers and an FBXW7 knockdown was shown to sensitize cancer cells to chemotherapy [26, 45]. Accumulation studies demonstrated that FBXW7 played a significant role in cancer cells by autophagy. For example, downregulated the expression of FBXW7 induced by high glucose activated mTOR signal, which led to diminished autophagy in renal mesangial cells [46]. Perifosine induces the degradation of key proteins in the mTOR axis through a GSK3/FBXW7-dependent mechanism in human lung cancer cells [47]. In addition, the relationship between miR-223 and FBXW7 has been studied in several cancers. In pancreatic ductal adenocarcinoma, miR-223 was reported to promote pancreatic cancer cell growth and invasion by targeting FBXW7 [48]. Moreover, the down-regulation of miR-223 could reverse the epithelial-mesenchymal transition (EMT) in gemcitabine-resistant pancreatic cancer cells [49]. In esophageal squamous cell carcinoma, the overexpression of miR-223 was found to promote tumor progression by inhibiting FBXW7-mediated regulation of the cell cycle [50]. In NSCLC, the miR-223/FBXW7 axis was reported to regulate doxorubicin sensitivity through EMT [20]. However, there are no existing reports on the miR-223/FBXW7 axis and the regulation of autophagy. In the present study, we have demonstrated for the first time that miR-223 can induce autophagy and enhance cisplatin-induced autophagy by targeting FBXW7.

## Conclusion

In conclusion, our findings indicate that miR-223 is a novel regulator of autophagy in NSCLC cells. Moreover, miR-223 was found to mediate autophagy by targeting FBXW7, which contributed to cisplatin resistance. Thus, the inhibition of autophagy by targeting miR-223-FBXW7 axis might provide a useful strategy for overcoming drug resistance in NSCLC.

## Supplementary information

**Additional file 1: Table S1.** Sequences used for siRNA and RT-PCR in the study.

**Additional file 2: Fig. S1.** Effect of cisplatin on the expression of LC3-I/II and SQSTM1 in NSCLC cells. (A) Western blot of LC3-I/II and SQSTM1 in NSCLC cells treated with the IC50 of cisplatin. (B) The relative expression of LC3-I/II and SQSTM1 protein was represented by calculating the grey value of the Western blot results. *p < 0.05, **p < 0.01 and ***p < 0.001.

**Additional file 3: Fig. S2.** miR-223 overexpression inhibited apoptosis in NSCLC cells. (A) Flow cytometry assay was performed to detect cell apoptosis in A549 and NCI-H1299 cells subjected to different treatment. *p < 0.05, **p < 0.01 vs. cisplatin.

**Additional file 4: Figure S3.** Effect of autophagy on cisplatin sensitivity and the expression of SQSTM1 in NSCLC cells. (A) NSCLC cells cultured in different concentrations of cisplatin were co-treated with 10 μM chloroquine. After 24 h, cell viability was determined using a CCK-8 assay. (B) NSCLC cells cultured in different concentrations of cisplatin were co-treated with 100 nM rapamycin. After 24 h, cell viability was determined using a CCK-8 assay. (C) Western blot of SQSTM1 in NSCLC cells treated with 10 μM chloroquine or 100 nM rapamycin.

**Additional file 5: Figure S4.** Western blot of FBXW7 in NSCLC cells.

## Data Availability

All data generated or analyzed during this study are included in this published article.

## Abbreviations

NSCLC: Non-small cell lung cancer
3′-UTR: 3′-untranslated region
P-MVs: Platelet-secreted microvesicles
ROS: Reactive oxygen species
ABC: ATP-binding cassette
EMT: Epithelial–mesenchymal transition
