# Supplementary material for: MiR-223 regulates autophagy associated with cisplatin resistance by targeting FBXW7 in human non-small cell lung cancer
Source: Cancer Cell Int. 2020 Jun 19;20:258. doi: 10.1186/s12935-020-01284-x (PMC7304223; doi:10.1186/s12935-020-01284-x)
Supplement: Supplementary file 1 — Additional file 1: Table S1. Sequences used for siRNA and RT-PCR in the study. [file 12935_2020_1284_MOESM1_ESM.docx]

Table S1. Sequences used for siRNA and RT-PCR in the study

| Targets | Primers |
| --- | --- |
| FBXW7 | Forward primer：CACTCAAAGTGTGGAATGCAGAGAC  Reverse primer：GCATCTCGAGAACCGCTAACAA |
| β-actin | Forward primer: TTCCAGCCTTCCTTCCTG  Reverse primer: CTTTGCGGATGTCCACGT |
| hsa-miR-223-3p | UGUCAGUUUGUCAAAUACCCCA |
| miR-223 mimics | sense: UGUCAGUUUGUCAAAUACCCCA  antisense: UGGGUAUUUGACAAACUGACAUU |
| miR-223 inhibitor | sense: UGGGGUAUUUGACAAACUGACA |
| FBXW7-Homo-1285 | sense: GCUGAAAGGACAUGAUGAUTT  antisense: AUCAUCAUGUCCUUUCAGCTT |
| FBXW7-Homo-1776 | sense: GGCAUACUAAUAGAGUCUATT  antisense: UAGACUCUAUUAGUAUGCCTT |
| FBXW7-Homo-2118 | sense: CGGGUGAAUUUAUUCGAAATT  antisense: UUUCGAAUAAAUUCACCCGTT |
