# Supplementary figures and images for: MiR-223 regulates autophagy associated with cisplatin resistance by targeting FBXW7 in human non-small cell lung cancer
Source: Cancer Cell Int. 2020 Jun 19;20:258. doi: 10.1186/s12935-020-01284-x (PMC7304223; doi:10.1186/s12935-020-01284-x)

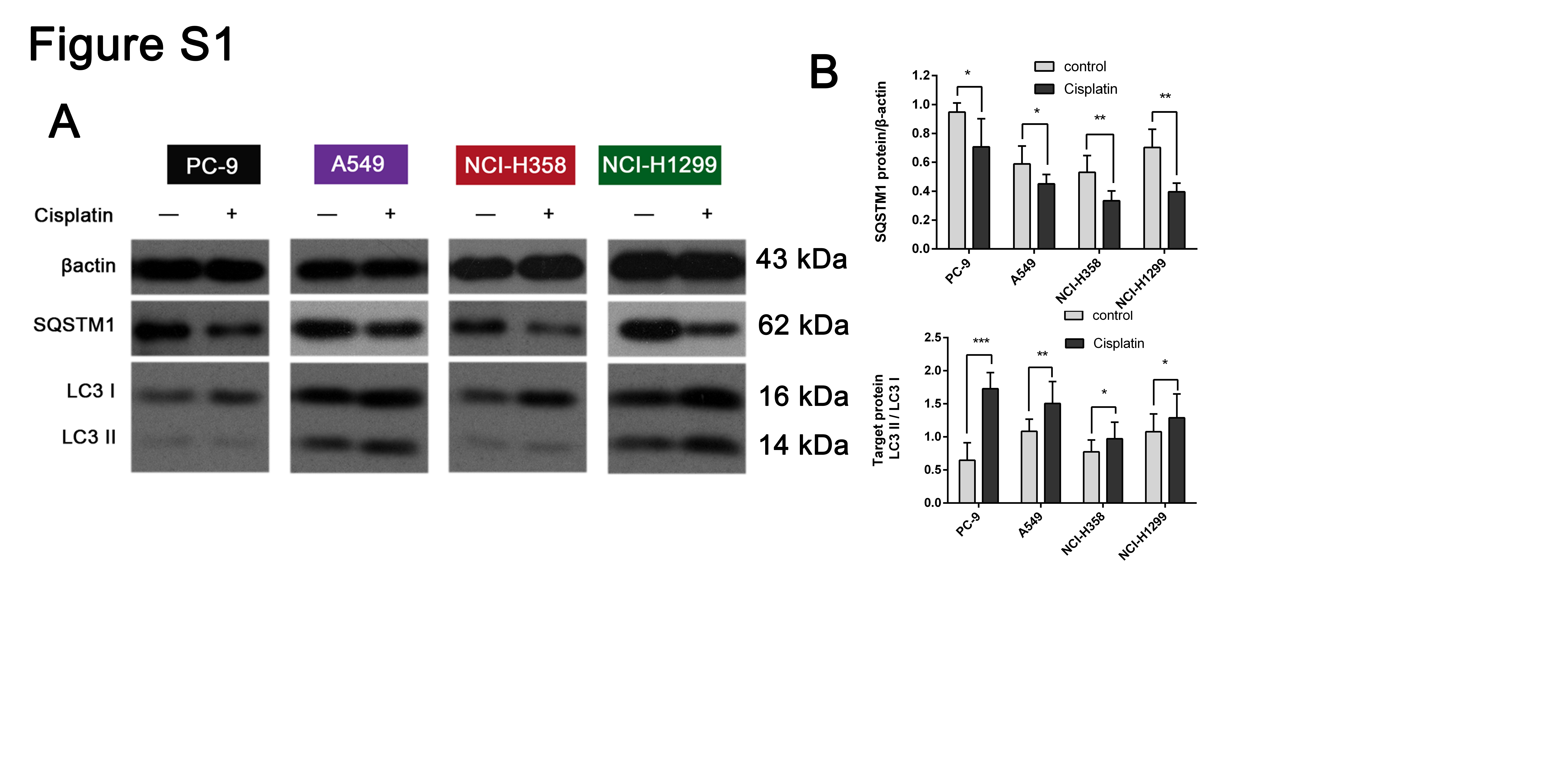

Supplement: Supplementary file 2 — Additional file 2: Fig. S1. Effect of cisplatin on the expression of LC3-I/II and SQSTM1 in NSCLC cells. (A) Western blot of LC3-I/II and SQSTM1 in NSCLC cells treated with the IC50 of cisplatin. (B) The relative expression of LC3-I/II and SQSTM1 protein was represented by calculating the grey value of the Western blot results. *p < 0.05, **p < 0.01 and ***p < 0.001. [file 12935_2020_1284_MOESM2_ESM.tif]

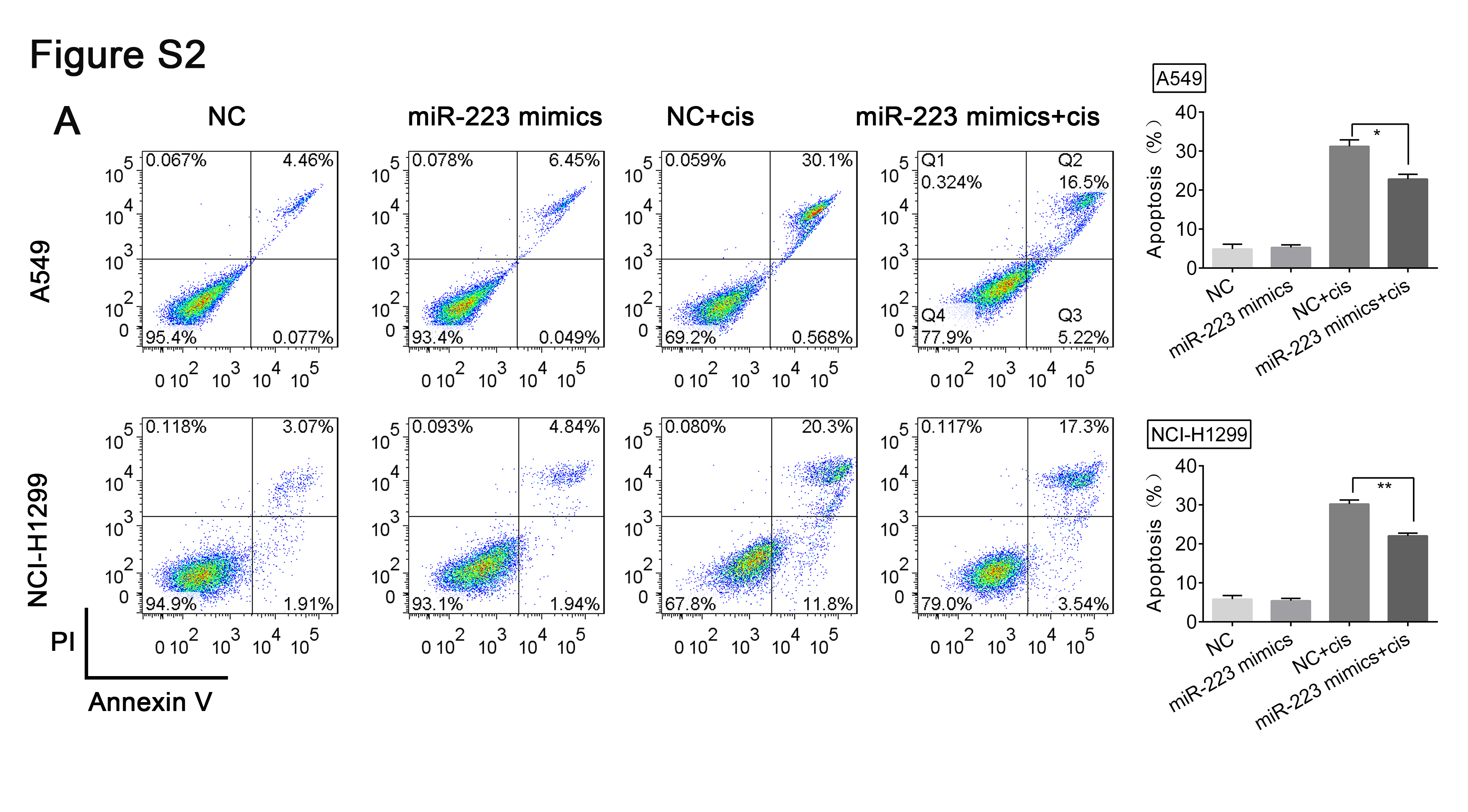

Supplement: Supplementary file 3 — Additional file 3: Fig. S2. miR-223 overexpression inhibited apoptosis in NSCLC cells. (A) Flow cytometry assay was performed to detect cell apoptosis in A549 and NCI-H1299 cells subjected to different treatment. *p < 0.05, **p < 0.01 vs. cisplatin. [file 12935_2020_1284_MOESM3_ESM.tif]

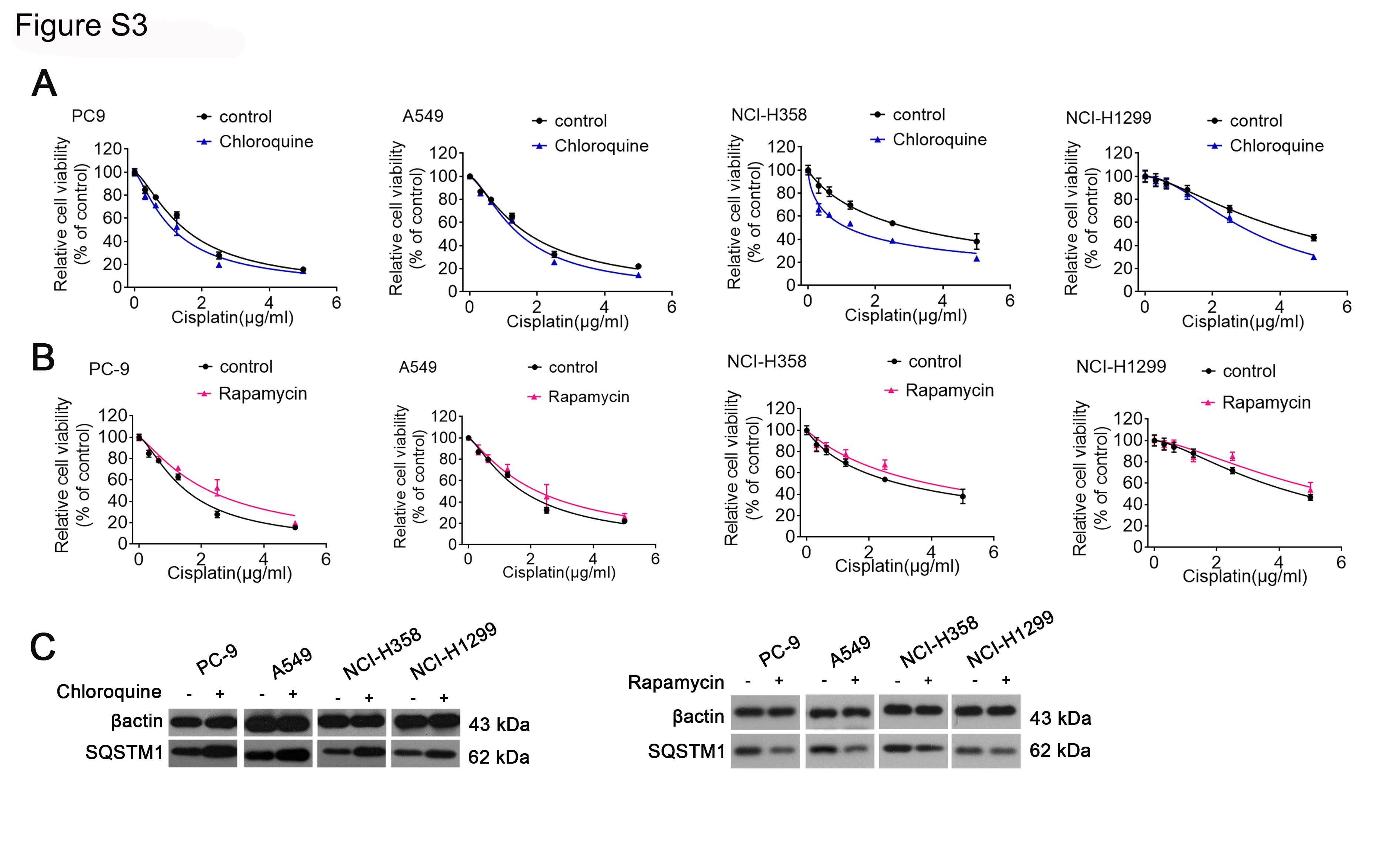

Supplement: Supplementary file 4 — Additional file 4: Figure S3. Effect of autophagy on cisplatin sensitivity and the expression of SQSTM1 in NSCLC cells. (A) NSCLC cells cultured in different concentrations of cisplatin were co-treated with 10 μM chloroquine. After 24 h, cell viability was determined using a CCK-8 assay. (B) NSCLC cells cultured in different concentrations of cisplatin were co-treated with 100 nM rapamycin. After 24 h, cell viability was determined using a CCK-8 assay. (C) Western blot of SQSTM1 in NSCLC cells treated with 10 μM chloroquine or 100 nM rapamycin. [file 12935_2020_1284_MOESM4_ESM.tif]

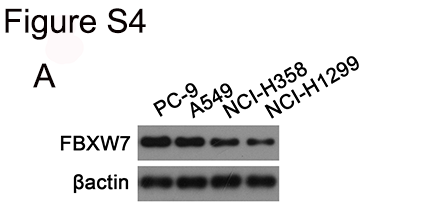

Supplement: Supplementary file 5 — Additional file 5: Figure S4. Western blot of FBXW7 in NSCLC cells. [file 12935_2020_1284_MOESM5_ESM.tif]
